# Supplementary material for: Are carbon emissions trading and green financial instruments synergistic? -Comprehensive quantitative research based on content analysis
Source: PLoS One. 2024 Mar 7;19(3):e0298601. doi: 10.1371/journal.pone.0298601 (PMC10919601; doi:10.1371/journal.pone.0298601)
Supplement: S1 Data — (ZIP) [file pone.0298601.s001.zip › Data/地级市Z_F工作报告.zip!/║■─╧/╘└╤⌠╩╨/└┤╫╘╣1⁄2╓┌║┼í╛┬φ┐╦╩2╛▌═°í┐_2007.docx]

各位代表：

现在，我代表市人民政府，向大会作政府工作报告，请予审议，并请市政协各位委员和其他列席人员提出意见。

一、过去五年工作回顾

市五届人大一次会议以来，全市上下在中共岳阳市委的领导下，坚持以邓小平理论和“三个代表”重要思想为指导，全面落实科学发展观，努力践行民本岳阳的执政和发展理念，“十五”计划圆满完成，“十一五”规划进展顺利，在加快富民强市进程中迈出了坚实步伐。

过去的五年，是经济实力大幅提升的五年。全市生产总值由2002年的394亿元增加到2007年的895.4亿元（预计数，下同），年均增长12.3 %，人均GDP达到16768元；财政总收入由28.2亿元增加到60.87亿元，五年实现翻番；三次产业结构调整为17.8:49:33.2。推进新型工业化取得重要进展，全市规模工业企业发展到942家，增加值年均增长19.7%，巴陵石化、长岭炼化、华能电厂、岳阳纸业、岳阳兴长等中央省属企业一批高科技大项目竣工投产，成为岳阳加快发展的重要支撑；岳阳经济开发区和县（市）区工业园快速发展，汨罗循环经济列入国家示范试点，云溪精细化工、华容纺织、临湘化工农药定为全省产业基地。现代农业水平提升，粮、棉、油整体进入国家长江流域优势产业带，生猪、水产养殖形成规模和特色，龙头企业发展到149家，涌现了正虹、岳泰、长康、福湘、义丰祥、巴陵油脂等优势品牌。市场消费拉力增强，社会消费品零售总额年均增长12.9%，年销售收入过亿元的市场增加到45家，旅游、文化和房地产业形成消费热点。县域经济发展提速，总量占全市的比重达到53%，县域特色更加明显，汨罗进入全省十强县（市）行列。

过去的五年，是改革开放扎实推进的五年。国有企业和国有资产管理体制改革步伐加快，农村综合改革不断深化，公共财政体系逐步建立。人事、投融资、金融保险等各项改革有序推进，全市金融机构各类存贷款余额分别达到458亿元和290亿元，每年举办的银企洽谈会支持了中小企业发展。全民自主创业行动深入开展，私营企业和个体工商户发展到12万多户，非公有制经济比重提高到52.5%。招商引资和项目建设支撑经济加速发展，五年累计实际利用内资402亿元、外资4.1亿美元，一批战略投资项目落地实施。口岸开放带动作用进一步增强，公共保税仓库投入营运，公路口岸获得批准，年进出口总额达到7.5亿美元。国际龙舟节、洞庭湖国际观鸟节、君山岛中国十大爱情故事评选活动等成为岳阳扩大开放的平台。

过去的五年，是城乡面貌显著变化的五年。累计完成全社会固定资产投资1072亿元，年均增长24.3%。洞庭湖风光带一、二期工程、环南湖旅游走廊等一批重点项目提升了城市品位，岳阳进入国家卫生城市、国家园林城市行列；各县城和小城镇面貌有新的改观，全市城镇化率达到45%。新农村建设迈出坚实步伐，志愿者行动和示范村建设深入开展，累计建成通乡公路1115公里、通村公路4100公里，新建沼气池8万口，农村生活用电同网同价面达到82.3%，实现了户户通电、村村通电话。交通能源建设快速推进，岳荣公路、临通公路、钟虹公路、湘阴湘江大桥、临资口大桥、注滋口大桥、输油和天然气管网等竣工，武广铁路客运专线岳阳段、荆岳长江大桥等进展顺利，新建了岳阳首座500千伏变电站。水利建设得到加强，五年完成投资53.3亿元，长江干堤全面加固，一批病险工程整治到位。节能减排力度加大，新上了一批排污治污设施和废弃物循环利用项目，加强了洞庭湖和南湖污染治理，全市森林覆盖率提高到35.8%，生态环境逐步改善。

过去的五年，是人民群众得到更多实惠的五年。全市城镇居民人均可支配收入达到13661元，农民人均纯收入达到4288元，五年分别净增5332元和1584元；城乡居民储蓄存款余额增加到308亿元，家庭财产普遍增多。各项支农惠农政策落到实处，五年全市累计投入支农资金73.4亿元，农民负担总额减少4.6亿元，对农民直接补贴11.8亿元。每年实施一批实事项目，五年新增城镇就业25.2万人，下岗失业人员再就业19万人，新增农村劳动力转移就业44.7万人；城镇职工养老、医疗、失业、工伤、生育保险参保人数达到182万人；改扩建乡镇卫生院132所、乡镇敬老院98所；计划生育奖励扶助、农村扶贫、残疾人帮扶、小街巷改造、重大安全隐患和污染源整治等都取得实效。

过去的五年，是社会建设全面进步的五年。各级各类教育迅速发展，素质教育在全国更具影响，在湖南率先实现职业学校人数超过普高人数。农村义务教育“两免一补”政策全面落实，农村中小学危房改造任务完成，中心城区新建了一批中小学校，教育布局更趋合理。湖南理工学院、岳阳职业技术学院、湖南民族职业学院扩建扩招提升了整体水平。成功战胜非典疫情，基本控制血吸虫病疫情，公共卫生和基本医疗服务不断健全。群众性文化、体育活动广泛开展，一批文艺创作精品获得全国精神文明建设“五个一”工程奖等奖项，岳阳被列为国家首批文化产业示范基地、中国楹联文化城市和国家语言文字达标城市。科技创新和招才引智成效明显。国土资源管理进一步加强。人口和计划生育工作保持全省先进。新闻出版、广播电视、民族宗教、外事侨务、社会科学、科普、统计、史志、档案、气象、双拥、人防和国防动员等各项工作都有新的进步。

过去的五年，是民主法制建设不断加强的五年。村民和居民自治巩固发展，社区建设力度加大。坚持依法治市，普法教育深入开展。行政管理体制改革不断深化，推进有错无为问责和政务公开，更加注重科学民主决策和依法行政。五年共承办人大议案、代表建议和政协建议案、委员提案2602件，办理行政复议案件158件，减少行政审批事项964项。工商、物价、质量技术监督、食品药品监督、海关、海事、检验检疫、烟草专卖、石油和食盐专营等在规范市场秩序中发挥了积极作用。深入开展反腐败斗争，加强审计监督和行政监察，查处了一批违法违纪案件。加强安全生产监管，有效遏制了重特大事故。畅通信访渠道，建立公共应急管理体系，加强社会治安综合治理，严厉打击各种违法犯罪，社会大局和谐稳定。

2007年是本届政府任期的最后一年。政府工作按照市第五次党代会和市五届人大五次会议确定的目标任务，务实创新，强力推进，岳阳已进入“全面提速、加快发展”的新阶段。

注重优势优先，推动又好又快发展。坚持把新型工业化作为富民强市的第一推动力，促进结构调整优化。工业经济实现“双百”目标，新增规模工业企业131家，年销售收入过亿元的企业增加到104家，新增了中天科技、天润化工两家上市公司；农业产业化发展加快，新增市级龙头企业39家；岳阳楼新景区竣工、湖南旅游节开幕式成功举办提升了人气指数，全年接待境内外游客1374万人次。全市主要经济指标创多年少有的高增幅，预计生产总值比上年增长14.5%，规模工业增加值增长21.2 %，全社会固定资产投资增长33.9%，实际利用内外资分别增长30.2%和21.9%，社会消费品零售总额增长18.5%，财政总收入增长23.3 %，城镇居民人均可支配收入增长16.5%，农民人均纯收入增长10.6%，人口自然增长率控制在6‰以内。

注重基础先行，构筑加快发展平台。把加强基础设施、基础产业和基础工作摆到更加突出的位置。基础设施加快推进，城陵矶（松阳湖）港区开工建设，岳汨公路、临鸭公路、铁桃公路、106国道平江段、202省道等改造工程启动实施，通过加大市财政配套投入，交通建设从国省道到乡村公路、城乡结合部公路全面展开；岳阳文化艺术会展中心和一批城镇建设工程进展加快。产业项目强势推进，岳纸40万吨项目和富士达无氟冰箱、建华管桩、金城陶瓷、兆邦陶瓷、龙舟铝业、中南黄金冶炼、华润雪花啤酒等一批招商引资项目开工建设，炼化扩能技改、华能三期、华容小墨山核电、君山风电、“洞庭湖国际公馆”大型旅游综合开发等重大项目前期工作取得新进展。科技创新等基础工作扎实推进，全市共取得科技成果25项，高新技术企业发展到94家，岳阳进入国家知识产权试点城市行列；与中南大学、湖南大学产学研合作全面启动，建立了5个省级创业基地；坚决关停65家中小造纸企业，实现了节能减排指标双下降。

注重改善民生，办好惠民实事项目。财政资金更多地投向民生领域，全年共安排支农、就业、社会保障、环境保护、社会事业等公共支出45.3亿元，增长34.8%。在全省率先建立“零就业”家庭就业援助和失地农民就业长效机制；中心城区城镇低保标准提高到200元，保障水平居全省第二，县（市）区提高到160元，8.2万人纳入农村低保；290万农民参加新型农村合作医疗；23.5万人享受库区移民扶持政策；新建廉租房603套、经济适用房6.1万平方米；岳阳县城铁山引水工程竣工通水，全市新解决23万城乡居民的饮水安全问题。

注重管理创新，提升政府服务水平。突出加大政策和制度创新的力度，完善了鼓励投资和规范收费、促进经济发展的财政奖励办法等政策措施，设立了工业园区建设发展、技术创新等引导资金，制定了市属国有企业改制总体方案和实施办法，理顺了城区财税管理体制，促成了市商业银行股权结构调整和经营效益提升。加大争创发展条件的力度，洞庭湖区特殊困难问题已纳入中央财政转移支付。加大优化经济环境的力度，市政府政务服务中心投入运行，社会治安综合治理考评居全省第二位，全市创业、创新、创优的氛围更加浓厚。

各位代表！岳阳五年来的成就，是市委总揽全局、正确领导的结果，是市人大依法监督、市政协民主监督和大力支持的结果，得益于多年来的扎实工作基础，凝聚着全市人民的辛勤劳动和不懈努力。在此，我代表市人民政府，向全市人民，向离退休老同志，向各民主党派、各人民团体和各界人士，向中央省属驻岳单位、驻岳解放军、武警官兵，向来岳投资者、建设者，向所有关心支持岳阳发展的海内外朋友们，表示崇高的敬意和衷心的感谢！

在看到成绩的同时，我们也清醒地认识到，我市经济社会发展还面临不少困难和问题。主要是：经济结构不优，区域经济竞争压力加大；国有企业改革滞后，体制矛盾仍然突出；节能减排任务艰巨，资源环境制约明显；食品药品安全、医疗服务、安全生产、社会治安等方面还存在群众不满意的问题，部分低收入群众生活困难。政府工作还不完全适应新形势、新任务的要求，社会管理和公共服务相对薄弱，政令不畅、效率不高、经济环境不优等问题和腐败现象依然存在。对此，我们一定高度重视，在今后工作中认真加以解决。

二、今后五年发展目标和工作任务

未来五年是岳阳加快发展的关键时期。党的十七大提出了实现全面建设小康社会奋斗目标的新要求，国家实施中部崛起战略，湖南促进“3+5”城市群发展，长株潭城市群、武汉城市圈获准为国家综合配套改革试验区。面对前所未有的新机遇和新挑战，面对岳阳人民过上更好生活的新期待，我们有责任、更有信心使岳阳跃上一个新的发展台阶，力争到2013年实现人均GDP翻两番。

今后五年，政府工作的基本思路是：深入贯彻党的十七大精神，以科学发展观统领经济社会发展全局，落实民本岳阳的执政和发展理念，坚持科学发展、好字优先，坚持改革开放、创新优先，坚持基础支撑、优势优先，坚持以人为本、富民优先，大力实施民本岳阳九项工程，加快实现“产业兴旺、城乡秀美、文化繁荣、民生殷实、社会和谐、走势强劲”的富民强市目标。

根据市第五次党代会的总体部署，今后五年主要奋斗目标是：

——经济实力明显增强。加快转变经济发展方式，建设现代工业大市、现代物流旺市、现代旅游热市、现代农业强市取得重大进展。到2012年，全市生产总值达到1830亿元，年均增长12%以上，人均GDP达到33000元；三次产业结构调整为10:51.4:38.6，非公有制经济比重提高到55%；财政总收入达到110亿元，年均增长13%；城镇化率提高到52%，县域经济实力不断壮大。

——生态环境明显提质。建设生态文明，整体谋划洞庭湖生态修复。五年全市万元GDP能耗下降20%，主要污染物排放总量减少10%，森林覆盖率达到36.8%，营造城乡山清水秀的宜居环境，努力创建国家生态园林城市。

——人民生活明显改善。健全合理有序的收入分配格局，全市城镇居民人均可支配收入达到21000元，农民人均纯收入达到6300元，年均分别增长9%和8%，中等收入者增多，人民群众拥有更多财富。

——文化建设明显加强。覆盖全社会的公共文化服务体系逐步完善，形成区域特色文化产业，社会文化生活更加丰富多彩，人民精神风貌更加昂扬向上。

——公共服务明显拓展。社会公平正义得到更好保障，人民群众享有更多更切实的民主权利。努力使人民群众学有所教、劳有所得、病有所医、老有所养、住有所居，社会安定有序，和谐社会建设迈出坚实步伐。

今后五年，政府工作的主要任务是：

（一）推进新型工业化。坚持走中国特色新型工业化道路，实施大项目带动、高科技支撑、低消耗发展，推动产业结构优化升级，加快建设现代工业大市。到2012年，全市规模工业增加值达到860亿元，年均增长16%以上。

突出提升层次，促进支柱产业强势发展。全力支持中央省属企业通过科技创新扩能升级、由大变强，增强核心竞争力。按照建设中南地区大型石化基地的目标，支持巴陵石化、长岭炼化尽快启动扩能技改项目，大力发展精细化工和化工新材料，力争原油加工能力突破1000万吨，石化产业年销售收入突破1000亿元，在区域经济中更具带动力和辐射力。按照建设全国大的造纸中心的目标，支持岳纸40万吨项目和丰利、丰隆纸业扩改项目加快建设，发展高档文化用纸，推进林纸一体化，带动印刷、包装和造纸机械等相关产业发展，力争造纸产业销售收入达到100亿元以上。按照建设电力能源大市的目标，支持华能三期、屈原生物质能发电项目加快建设，继续做好华容小墨山核电、平江抽水蓄能发电、君山风电、汨罗火电、华能岳阳第二电厂等项目前期工作，“五电”并举组合开发。发挥农产品加工基础较好的优势，扩大产业规模，提升科技含量，创建大品牌，力争食品加工产业销售收入达到300亿元以上。

突出自主创新，培育新兴产业扩张发展。抓住生物医药产业空间布局尚未定型的有利时机，支持科伦药业、景达生物等扩大市场份额，加快发展中医药产业，引进和培育一批拥有自主知识产权的优势企业，建设生物医药产业基地。支持汨罗循环经济把产业规模、品牌效应和示范作用做大，建成全国环保科技型再生资源回收、利用、加工示范基地；引导园区和更多企业循环式生产，扶持发展环保产业。支持特种电机、汽车空调、农用机械、潜泵、电磁铁等优势传统品牌改造提升，促进在岳军工企业扩大民品生产，建设先进制造业基地。强化政府对自主创新的支持和服务，与科研院所形成产学研战略联盟，发挥信息技术的倍增和催化作用，加快发展高新技术产业，力争增加值占规模工业的比重提高到35%以上。实施品牌战略和知识产权战略，大力支持企业创品牌，力争进入国家知识产权示范城市行列。

突出园区建设，引导中小企业集群发展。按照产业集群基地、科技创新平台、优先发展区域的要求，进一步加快工业园区建设。岳阳经济开发区着力发展高新技术产业，创新管理体制，力争跻身国家级开发区行列。县（市）区工业园进一步做大做强主导产业，形成精细化工、先进制造、生物医药、绿色食品、陶瓷建材、纺织、饲料等特色产业聚集区，力争园区规模企业、工业增加值和税收均达到总量的一半以上。实施中小企业成长工程，承接大厂产业链延伸扩张一批，加快国有企业改制催生一批，推动全民自主创业新上一批，引导关联度高的中小企业向优势企业靠拢、向工业园区集群。到2012年，全市规模工业企业达到1500家，形成一批销售收入过10亿元、过50亿元、过100亿元的优势企业，构建“大树成林、枝繁叶茂”、各类企业同生互促的工业生态。

突出港口优势，推动沿江产业带快速发展。把打造港口带动型的沿江产业带作为岳阳优势优先发展的突破口。充分利用岸线资源，以城陵矶港区为核心港，建设现代化港口群。到2012年，全市港口货物吞吐量达到4500万吨、30万标箱以上。重点加快城陵矶（松阳湖）港区建设，完成口岸设施改扩建，科学规划港区产业布局。高水平建设城陵矶物流园，与太阳桥、枫桥湖物流园融合，与长江下游大型枢纽港对接，努力把岳阳建成长江中游重要的区域物流中心；引进战略投资者临港布局大运量、大耗水工业项目，整合发展重化工业、造船等产业，大力发展加工贸易，争取开辟国家级出口加工区，形成港口、物流、保税、工业“四位一体”发展的新型港口经济。同时，新建改造漕溪港、营田、鹿角、鸭栏、塔市驿、荆江门等30个1000吨级以上码头，与县（市）区工业园连通，建设沿江沿湖经济走廊。

（二）推进新农村建设。始终把解决好农业、农村、农民问题作为重中之重，突出加强农业基础建设，积极促进农业稳定发展、农民持续增收，形成城乡经济社会发展一体化新格局。

加快农业产业化进程。用新型工业化理念推进现代农业。充分发挥农业的多种功能，发展规模型、标准型农业。巩固提升粮、棉、油、菜、茶等优势产业带，加快优质农产品基地建设，增创10个国家级农业标准化示范区。重视发展特种养殖业、园艺业和乡村旅游业，加快培育一批特色明显的专业村、专业乡镇，争取开辟“海峡两岸农业合作试验区”。充分发挥龙头企业的带动力量，发展加工型、品牌型农业。突出大宗农产品深加工、主导产品精加工和副产品综合利用，继续抓好饲料生猪、优质稻米、棉纺等10大产业链建设，培育几个销售收入过30亿元、50亿元的龙头企业，打造全国农产品加工示范基地。充分发挥市场的引导作用，发展安全型、生态型农业。改造提升农产品批发市场，加快建设农村现代流通服务网络，落实绿色通道政策。提高农业机械化装备水平，健全农业技术推广、农产品质量安全、农业生态安全和动植物疫情防控体系，打造岳阳农业的绿色品牌。

加大支农惠农政策落实力度。全面落实粮食、良种、农机购置、退耕还林、农资综合补贴以及农业保险、库区移民扶持等各项政策。按照“三个明显高于”的要求，加大“三农”投入力度，积极探索涉农项目资源整合的新办法，把基础设施建设和社会事业发展的重点转向农村。五年改造建设通乡公路500公里、通村公路5000公里以上，到2012年全市所有乡镇和98%以上的建制村通水泥路；全面完成第三期农村电网改造，推进电气化乡村试点工作，实现城乡用电同网同价；解决78万农村人口饮水安全问题；农村沼气入户普及率达到30%以上；实现广播电视“村村通”。继续深入开展志愿者行动，发挥示范村、示范片的引导带动作用，使新农村建设普惠千家万户。

深化农村综合改革。积极稳妥推进乡镇机构、农村义务教育和县乡财政管理体制改革，推进国有农场税费改革、洞庭湖区农民负担综合改革和集体林权制度改革。坚持农村基本经营制度，按照依法自愿有偿原则，健全土地承包经营权流转市场，发展多种形式的适度规模经营。加快征地制度改革，妥善解决好失地农民的安置问题。继续做好农村减负工作。积极化解乡村债务，防止新债，保障基层政权正常运转。

提高农民创业致富能力。充分发挥广大农民建设新农村的主体作用。加大“阳光工程”等培训支持力度，提高农民科学种养技术水平和转移就业能力，培育有文化、懂技术、会经营的新型农民。大力发展劳务经济。提高农村公共服务人员能力。积极发展运作规范的农民专业合作组织，培育种养大户、农村经纪人和农民致富带头人，带动更多的农民发家致富。

（三）推进新型服务业。适应经济增长依靠消费投资出口协调拉动、依靠三次产业协同带动的新趋势，大力发展新型服务业，实现第三产业跨越发展。

发展特色鲜明的旅游产业，做大支柱。依托品牌造势，使岳阳更吸引人。发挥“天下水、天下楼、天下岛”品牌和岳阳楼景区的影响力，用市场手段办好国际龙舟节、洞庭湖国际观鸟文化节和君山岛爱情主题活动，打造“热游岳阳”的城市名片。抓住端午节定为法定假日的契机，让岳阳龙舟文化发祥地更引人注目。建好休闲基地，使岳阳能留住人。加快“洞庭湖国际公馆”等大型旅游综合开发项目建设，整合开发平江红色旅游、张谷英民俗文化村、屈子祠等特色旅游资源，发展乡村休闲旅游，推动景区晋级、宾馆评星、导游创优，提升旅游综合服务能力，实现3—5日游。创新经营方式，使岳阳融入大旅游格局。加强与旅游经销商、著名景区和热点线路的协作，加快旅游基础设施建设和旅游产品开发，努力创建中国最佳旅游城市。到2012年，全市旅游业比重提高到8%以上。

培育持续旺盛的生活需求，拉动消费。适应消费升级，把增加居民消费特别是农民消费作为重点，不断拓宽消费领域。推动商贸流通业提升水平，构建中央商务区、商业步行街、大型购物中心、连锁超市、专业批发市场等多层面服务网络。推动房地产业健康发展，科学引导住宅房地产，积极调整投资经营性房地产，大力发展公益性房地产，使房地产业成为新的发展阶段一个重要支柱产业。推动社区服务业形成网络，引导文化娱乐、体育健身、教育培训、大众餐饮等进行产业化运作，促进家政、养老、保健等向企业化发展，不断培育新的消费热点。

建设开放有序的市场体系，激活要素。大力发展生产性服务业，进一步完善资本、土地、产权、技术、人才和劳动力等要素市场，扶持一批优良企业上市融资，培育“会展经济”、“楼宇经济”、“第三方物流”等新的增长点。大力发展金融保险业，加强同金融机构的沟通协作，支持金融服务创新综合改革试点，推进市商业银行改革重组和加快发展，扩大保险业务，改善金融生态环境，努力创建金融安全区。大力发展中介服务业，促进认证、咨询、代理、担保等各类中介机构和行业协会加快发展、规范运作。整顿和规范市场秩序，加快社会信用体系建设，加强价格监控和市场监管，切实抓好产品质量和食品、药品安全，坚决打击制假售假、商业欺诈、偷逃骗税、传销等违法活动，让人们放心消费、安全消费。

（四）推进新型城镇化。努力把岳阳建成长江中游现代化工业、港口、旅游城市和水秀山清的宜居城市，引领带动城镇发展，培育区域经济增长极。

加快拓城提质，增强城市吸纳带动功能。以战略眼光拉开城市主框架，做大做美中心城市。城市发展方向总的是“东扩、北靠、西联、南延、中提”：“东扩”——实施“前沿发展”，抢抓国家重大交通项目建设的机遇，重点推进武广客站新城区建设，规划建设随岳、岳常高速公路与城市的对接区域，建设最具现代气息的新型服务和高新产业区。“北靠”——实施“沿江发展”，重点推进城陵矶（松阳湖）港区建设，规划改造岳云大道，建设港口带动型沿江工业区。“西联”——实施“跨湖发展”，重点抓好洞庭湖大桥桥西区域开发，建设生态旅游和现代农产品加工区。“南延”——实施“对接发展”，重点依托大型综合旅游项目，沿洞庭湖岸线向南开发，建设休闲度假区。“中提”——实施“精品发展”，重点推进中心城区改造提质，建设宜居、商务、旅游多功能聚集区。续建洞庭湖风光带三期工程、环南湖旅游走廊和岳阳文化艺术会展中心，建设规划展览馆和洞庭湖博物馆；加快实施“城中村”和旧城改造，形成特色风貌街区；加快实施沿湖老厂区“退二进三”、“腾笼换鸟”；加强南湖、芭蕉湖、东风湖等内湖的保护和开发，着手规划恢复流水环城的生态景观。力争城市建成区拓展到120平方公里，功能分区和产业布局更加完善，充分展示岳阳的湖光山色美、古今交融美、现代动感美。

加快构建城镇体系，支撑县域经济快速发展。2个县级市和4个县城按20—30万人的目标规划建设，支持市区周边、市域周边40个小城镇加快发展，建设一批重点示范镇和特色产业镇，培育承载农民转移、要素流动、消费聚集、产业发展的更多节点。把加快县域经济发展摆到更加突出的位置，进一步落实各项政策和措施，健全市财政对县（市）的奖励机制，建立科学的评价考核体系，形成市直部门多支持、多服务的强大合力。支持县（市）发挥比较优势，大力发展园区工业和乡镇企业，培育特色产业。力争县（市）经济总量分别迈上100亿元、150亿元的台阶，财政总收入迈上6亿元、12亿元的台阶。

加快创新运作方式，提升城镇管理经营水平。进一步理顺管理体制，明确市、区、街道三级工作责任；加强规划执法，坚决控制违法建设；运用科技手段，推进数字化城管；加大市场运作，争取发行城建债券，提高城镇土地、特许权和无形资产经营效益，对市政维护、环卫保洁、园林绿化等引入竞争机制。进一步完善城镇基础设施，社会事业和社区服务设施同步规划、预留空间，扩大公共绿地面积，实施公交优先。努力实现由集中整治脏乱差向长效管理、塑造城市形象转变，由注重城镇建设向整体联动、促进城乡秀美转变。

（五）推进基础设施建设。实现又好又快发展，必须有效扩大投资，多干打基础、利长远的事。

构建内外通畅的综合交通运输枢纽。支持武广铁路客运专线岳阳段、岳常和随岳高速公路如期建成，争取启动京珠岳长复线和荆岳铁路建设，构建“一横三纵”高速公路网；改造升级460公里国省道干线公路，建成汨罗江、鲇鱼须、梅田湖、柳林江大桥，实现湖区干线公路“渡改桥”；争取国家和省里更加重视长江黄金水道建设，加强航道疏浚，建成洞庭湖水上应急救援指挥中心。全面打开对外通道，形成多条高速公路交汇、多条铁路干线接轨、多层次水运联网的中部地区重要的综合交通运输枢纽。提高能源供应的保障能力，加快建设平江、云溪、荣家湾220千伏变电站等工程。加强信息设施建设，推广数字电视，提高邮电通信服务水平，加快经济社会信息化。

建设人水和谐的现代水利体系。树立治水新理念，加快建立完善的防洪减灾、供水保障、水环境保护和水务管理体系。积极争取国家加大洞庭湖综合治理，重点抓好湖区薄弱堤段建设、山丘区中小型水库除险加固、中型灌区续建配套以及大型排涝泵站更新改造。抓好层山安全区试点工程和全国节水型社会建设试点，完成平江、华容、临湘、汨罗城区引水工程建设，规划实施铁山水库外引水源工程。加快建设各类抗旱基础设施，解决好生活生产用水。

打造资源节约型、环境友好型的地域特色。严格守住保护耕地、改善生态、节能减排、保障民生“四条底线”，努力建设“生态岳阳”。实行最严格的土地管理制度，推进节约集约用地，盘活存量土地，完善土地储备制度，保障民生项目和符合国家产业政策项目的用地需求。把节能减排纳入经济社会发展综合评价体系，落实工作责任制。坚决淘汰低效高耗企业，坚决拒绝环保不达标项目，坚决杜绝低水平重复建设，促进产业优化；建设开发区、云溪工业园等污水处理厂，加快各县城污水、垃圾处理设施建设，开展污染源普查，促进达标排放；加强洞庭湖和各流域综合治理，加强东洞庭湖湿地、铁山库区等生态功能区建设，加强森林、矿产等自然资源保护，控制农村面源污染，促进生态修复。全社会积极行动，共同保护我们赖以生存的环境。

（六）推进改革开放。今后五年改革开放必须迈出更大步伐，为加快富民强市提供强大动力和体制保障。

坚定不移推进各项改革。力争在关键领域和重要环节取得突破，形成“活力岳阳”的新格局。加快国有企业改革和非公有制经济发展，推进经济主体多元化。以更大的决心、更硬的措施推进市属国有企业改制，支持中央省属企业主辅分离、辅业改制、主业扩张，进一步完善国有资产监管体制。按照“民营岳阳”工程的要求，毫不动摇地鼓励、支持、引导非公有制经济发展，推进公平准入，改善融资条件，破除体制障碍，平等保护物权。大力推动全民创业，培育一大批有创新精神和社会责任感的企业家，使非公有制经济在富民强市进程中发挥更大作为。加快财税体制改革，推进公共服务均等化。应对省以下财政体制改革的新形势，着力扶持财源建设，加大综合治税力度，促进财政增收提质。从2008年起实施新的城区财税管理体制，促进事权与财权相统一。建立财政预算绩效评价体系，把财政资金更多地投向民生领域、公益事业和困难群体。加快行政管理体制改革，推进行政运转高效化。着力转变政府职能，全面推行人事制度改革和事业单位分类改革，严格控制编制，降低行政成本。坚决把不该由政府管理的事交给市场，在公共服务领域更多地引入市场机制。

全方位扩大对外开放。岳阳是沿江首批对外开放城市，要增创经济全球化条件下的开放新优势。打破行政区划局限，全方位对接发展。积极应对长株潭城市群和武汉城市圈定为国家综合配套改革试验区的机遇和挑战，实行双向对接，力求南北逢源、乘势崛起。发挥地域优势主动对接，湘阴、汨罗争取进入长株潭改革试验区政策范围，平江、岳阳县、屈原主动融入长株潭接受辐射，临湘、华容在湘鄂边界区域凸显特色、赶超发展；发挥流域优势同质共建，联手加强江湖治理，加快建设“两型”社会；发挥港口口岸优势承接产业，吸纳大运量、大耗水项目落户和大物流中转；发挥中间接点优势打开进出通道，谋求与“泛珠三角”、“长三角”、国内外友好城市等更大范围的区域经济合作。调动一切积极因素，大力度招商引资。进一步加大实施“置业岳阳”工程的力度，各级领导、职能部门和驻外地机构都要有抓招商的闯劲、钻劲和韧劲。盯住跨国公司、中央企业、上市公司、大型民营企业等战略投资者，持续推进大项目和产业链招商。创新招商方式，积极推动企业主体招商、中介专业招商，在招商活动中讲求实效。努力开拓国际市场，走出去参与竞争。立足以质取胜，促进加工贸易转型升级，承接和发展服务外包，促进更多的企业、产品和劳务走向国际市场，实现开放型经济发展的新突破。

坚持不懈优化经济环境。把政府转变职能、推动发展的着力点放在优化经济环境上。一切为企业基层着想。增强政府及各部门服务经济的意识，行政运作程序、制度安排和工作方式都要想企业所想、急基层所急，主动适应市场主体，为投资者提供优质高效服务。一切按公正公平执法。推进行政执法体制改革，依法规范行政处罚自由裁量权，加大经费保障力度，从源头解决多头执法、趋利执法、“收费养人”的问题。全面推进政务公开和电子政务，减少和规范行政审批，促进政务服务中心规范高效运作，让权力在阳光下运行。一切凭制度承诺问责。按照“诚信岳阳”工程的要求，进一步健全优化经济环境的各项制度，政府各部门向社会公开服务承诺。通过公开讲评、广泛监督，促进制度落实和承诺兑现，对行政不作为、乱作为、扰乱经济环境的坚决问责查处，对不合格的公务员按规定坚决辞退。

（七）推进文化和社会建设。在经济发展的基础上，更加注重文化建设和社会建设，提升公共服务水平。

推动文化繁荣。围绕建设社会主义核心价值体系，大力实施以“文明岳阳”为主题的精神文明建设工程，加大创建全国文明城市的力度。在创意、管理、运作上下工夫，发展大型文化产业项目，加快文化产业基地建设，扶持公益性文化事业，力求出精品、出人才、出效益。在传承中大力培育现代工商文化，倡导形成创新创业、敢冒敢富、开放开明、守信守法的社会氛围。积极发展新闻出版、广播影视、文学艺术、文物、史志、档案事业，鼓励社会科学界发挥思想库作用。加强全社会特别是青少年思想道德建设，加强科学普及和普法教育，提高全民素质。广泛开展全民健身运动，提高竞技体育水平，加快市体育中心等场馆建设，积极申办省第十二届运动会。建设市档案馆、市广播电视中心和各乡镇综合文化站，推进文化资源信息共享工程、农村电影放映工程、农家书屋工程等建设，积极发展社区文化和企业文化，使人民群众基本文化权益得到更好保障。

建设教育强市。坚持优先发展教育，促进义务教育均衡发展，加快普及高中阶段教育，大力发展职业教育，提高高等教育质量。重视学前教育，关心特殊教育。坚持教育公益性质，加大财政对教育投入，保障经济困难家庭、进城务工人员子女平等接受义务教育，基本化解农村义务教育“普九”债务。鼓励和规范社会力量兴办教育。加强教师队伍建设，优化城乡教师队伍结构。巩固发展素质教育，继续保持高中阶段普职招生人数1:1，做强岳阳教育品牌。大力培养和引进人才，造就一大批经济、科技领军人才和高技能人才，开创人才辈出、人尽其才新局面。

完善社会管理。健全充满活力的基层群众自治机制，推进村务、厂务公开。重视加强和谐社区建设，积极改善社区工作条件，支持社会组织参与社会管理。全面执行民族、宗教、侨务和对台政策，发挥工会、共青团、妇联、科协等人民团体的桥梁和纽带作用，发挥宗教界人士和信教群众在促进经济社会发展中的积极作用。认真实施妇女、儿童发展纲要，更加关爱“留守儿童”，进一步加强老龄工作，加快发展残疾人事业。完善信访制度，强化信访责任，促进信访事项及时、依法、就地解决。加强行政复议和人民调解工作。坚持安全发展，全面落实安全生产责任制，切实抓好重点行业和领域专项整治，坚决遏制重特大事故。完善应急管理机制和平台，有效应对自然灾害、事故灾难、公共卫生和社会安全事件。深入开展“平安岳阳”创建活动，加强社会治安综合治理，加强城乡社区警务工作，依法防范和打击违法犯罪活动，切实维护社会稳定。加强国防教育和国防后备力量建设，深入开展双拥共建，巩固发展军政军民团结。

（八）推进改善民生。始终把实现好、维护好、发展好人民群众的根本利益作为政府一切工作的出发点和落脚点。

实施扩大就业的发展战略，使更多的劳动者成为创业者。加强政府引导，统筹城乡就业，积极支持自主创业和自谋职业。进一步完善面向困难群众、弱势群体、失地农民的就业援助制度，及时帮助“零就业”家庭解决就业困难。健全面向全体劳动者的职业教育培训制度。发展和谐劳动关系，全面推行劳动合同制度，依法维护劳动者权益。进一步规范收入分配秩序，着力提高低收入者收入，创造条件让更多群众拥有财产性收入，促进共同富裕。

加快建立覆盖城乡的社会保障体系，让人民群众享有基本生活保障。以社会保险、社会救助、社会福利为基础，以基本养老、基本医疗、最低生活保障制度为重点，以慈善事业、商业保险为补充，加快完善社会保障体系。进一步扩大城镇职工养老、医疗、失业、工伤、生育保险覆盖面，提高非公有制企业、灵活就业人员、农民工参保率。全面推进城镇职工、城镇居民基本医疗保险和新型农村合作医疗制度建设。完善城乡低保、医疗救助、救灾救济制度，逐步提高保障水平。健全廉租住房制度，加快解决低收入家庭住房困难。以平江为重点实施扶贫整村推进。采取多种方式充实社会保障基金，强化社会保障基金、住房公积金等监管。

建立基本医疗卫生制度，为人民群众提供良好的医疗卫生服务。坚持公共医疗卫生的公益性质，强化政府责任和投入，建设覆盖城乡居民的公共卫生服务、医疗服务、医药保障、药品供应保障体系。加强农村三级卫生服务网络和城市社区卫生服务体系建设，建设市公共卫生中心，做好重大疾病防控工作，深入开展爱国卫生运动。深化医药卫生体制改革，提高医疗服务质量，努力解决好广大群众关心的看病就医问题。坚持计划生育的基本国策，稳定低生育水平，提高出生人口素质，统筹解决出生人口性别比偏高问题，加强流动人口服务和管理，落实奖励扶助政策。加快推进和谐社会建设，让人民共享发展成果，让城乡居民特别是困难群众都能享受到公共财政的阳光。

三、扎扎实实做好2008年工作

2008年是全面贯彻落实党的十七大作出的战略部署的第一年，也是新一届政府的开局之年，做好2008年工作至关重要。全市经济社会发展预期目标是：生产总值增长12%以上，经济总量迈上1000亿元台阶；财政总收入增长13%，全社会固定资产投资增长20%，社会消费品零售总额增长15.5%，城镇居民人均可支配收入增长9%，农民人均纯收入增长8%，居民消费价格涨幅控制在5%以内，人口自然增长率控制在6.2‰以内，各项社会事业都有新的进步。实现上述目标，必须深入贯彻中央和省委经济工作会议精神，积极应对世界经济发展的新趋势，把握国家宏观调控政策的新要求，坚持好字优先、好中求快、稳中求进、紧中求活，加力加速发展，实现开局之年的新突破。

（一）抓重要规划编制。根据长株潭城市群、武汉城市圈定为国家“两型社会”综合配套改革试验区的新情况，重新审视岳阳的各项规划，在主动融合中加强规划修订和编制。重点完善全市产业发展规划、区域发展规划、民生项目专项规划；完成全市土地利用总体规划、城市总体规划和市域城镇体系规划，两年内城市控制性详规覆盖率达到100%；全面开展第二次经济普查和土地调查，尽快形成各类规划定位清晰、功能互补、有效衔接的规划体系。加大五年目标任务和规划的落实力度，按年度层层分解，量化到部门，激励政策、保障措施同步跟进，确保各项工作一开局就按照科学发展的要求有序、强力推进。

（二）抓重点工作突破。紧紧抓住经济发展的关键领域，力求率先取得明显成效。突出经济提速，一产业主要是夯实基础、加快产业化，争取全市规模农产品加工企业达到320家，新推出1—2家在全省、全国有影响的优势企业；二产业主要是优化结构、提高产业发展水平和经济效益，全市新增100家规模工业企业，新增10家税收过1000万元的企业；第三产业主要是加速发展、提升层次，全市旅游收入增长30%以上，培育和引进一批大型物流企业。突出国企改制，各职能部门把改革作为头等任务，切实担负起责任，以存量资产和市场品牌换取改制资金，基本完成28家市属国有企业改制任务。突出招商引资，进一步完善考评办法，资金到位看银行、开工建设看现场、设备水平看台账、投产见效看税收，力争引进内资120亿元、外资1亿美元以上。突出节能减排，继续开展万吨耗能企业节能行动，加快实施10大减排工程，确保完成节能减排约束性指标，促进生态改善和可持续发展。

（三）抓重大项目建设。将2008年作为“招商引资和项目建设年”，突出抓好产业发展大项目、基础设施大工程，着力推进50个重点项目建设和20个重点项目前期工作。产业发展方面，加快岳纸40万吨项目和工业园区在建项目建设，争取炼化扩能技改、华能三期等年内开工；交通建设方面，建成岳荣新公路二期工程、漕溪港码头等项目，加快城陵矶（松阳湖）港区一期工程、通港路、各县（市）区干线公路等建设；城镇建设方面，完成岳阳文化艺术会展中心、青年中路等建设，加快武广客站和巴陵东路、洞庭湖风光带三期工程、县城饮水工程等建设。全面把握宏观调控的方式、方向和重点，用活用好可用资源，积极化解资金、用地、环保压力，为项目建设和长远发展赢得更大空间。2008年我市将举办第二届湘商大会、中国（湖南）红色旅游文化节开幕式暨平江起义80周年纪念活动、北京奥运火炬传递等大型活动，努力办出特色、办出水平、办出实效，进一步展示岳阳开放形象，增创新的发展条件。

（四）抓民生实事落实。面向困难群众和弱势群体，办好10件实事。1、全市新增城镇就业4.2万人、下岗失业人员再就业2万人，新增农村劳动力转移就业7万人。2、改造建设2000公里通乡通村公路和一批农村客运站，全面完成中心城区城乡结合部117公里道路改造，开展创建“文明路”活动。3、城镇职工五大社会保险共新增参保人数10万人，全面启动城镇居民基本医疗保险、覆盖80万人，农村低保面扩大到3%。4、新增廉租房1000套、经济适用房12万平方米，实行廉租房租赁补贴，基本解决1万户最低收入家庭住房困难。5、提高农村家庭经济困难寄宿生生活费补助标准，向农村义务教育阶段所有学生免费提供教科书，免除城市义务教育学杂费。6、改扩建19所乡镇卫生院，新增21个城市社区卫生服务中心和22个社区卫生服务站。7、改扩建乡镇敬老院50所，在全省率先实现“乡乡有敬老院”，实施农村爱民安居工程建房1200栋，建设市残疾人康复就业综合服务中心和市儿童福利院。8、新增通广播电视的自然村900个以上。9、解决10万农村人口的饮水安全问题，新建农村沼气池2.2万口。10、抓好18处重大安全隐患和12处重点污染源整治。对实事项目，我们一定办好办实，如期如质向各位代表交账，让人民群众满意。

四、加强政府自身建设

实现今后五年的发展目标，加强政府自身建设至为关键。政府工作人员必须毫无保留地忠诚于党和人民、忠诚于事业，努力建设对人民负责、让人民满意的政府。

继续解放思想，增强工作前瞻性和创造性。坚持用党的十七大精神武装头脑，使解放思想真正体现在解决影响发展的实际问题上。统筹全局，坚持牢牢扭住经济建设中心，不为任何风险所惧、不被任何干扰所惑，有所为有所不为，从繁杂事务中解放出来；谋划发展，坚持把岳阳放在大格局中比较分析，增强忧患意识和竞争、进位意识，用世界眼光和开放理念应对机遇挑战，从自我封闭中解放出来；推进工作，坚持对全局性、根本性问题进行深入研究，敢于突破“惯例”、敢于超越自我，创造性地提出政策措施，从传统思维定势中解放出来。始终保持昂扬向上的精神状态和工作激情，以思想的不断解放推动事业的不断发展。

坚持依法行政，推进决策民主化和科学化。严格按照法定权限和程序行使权利、履行职责、接受监督。认真落实人大及其常委会的决议决定，定期报告重要工作；尊重和发挥人民政协的职能，广泛听取各民主党派、工商联、无党派人士和各人民团体的意见；认真办理人大议案、代表建议和政协建议案、委员提案。加强政府法制工作，提高政府依法管理社会的能力。完善集体决策、专家咨询、公开听证、社会公示制度，保障人民群众的知情权、参与权和监督权。充分相信和依靠人民群众，把关心群众与引导群众很好地统一起来，最大限度地集中人民群众的智慧和力量，共同建设我们美好家园。

着力提高效能，强化政府执行力和公信力。加快政府管理方式创新，进一步健全行政责任体系，实行更加严格的目标管理、更加严格的绩效考核、更加严格的督查督办、更加严格的责任追究。建立部门间内部协调沟通机制，减少管理环节，重点解决职能交叉、责任不清、事权分离和管理真空等突出问题。健全政府组成部门向政府全会报告工作制度。今后凡因工作失误或失职造成公共利益受到重大损失的，都要公开道歉，勇于承担责任，并启动行政问责，这是建设责任政府的基本要求！

加强廉政建设，塑造公务员队伍新风貌和新形象。严格履行廉政建设责任制，更加注重治本、注重预防、注重制度建设，把廉政的要求落实到每一个行政业务工作岗位，落实到每一项行政权力行使过程。支持监察、审计部门依法独立履行监督职责。坚决纠正损害群众利益的不正之风，坚决查处违法违纪案件。按照“务实岳阳”工程的要求，认真实施《公务员法》，努力建设一支学习型、高素质、职业化的公务员队伍，把精力集中在想干事上，把本领体现在会干事上，把目标锁定在干成事上，全心全意为人民服务。

各位代表！历史是人民创造的，任何困难都不能改变岳阳人民创造美好幸福生活的坚定信念，任何挑战都无法阻挡我们实现富民强市目标的前进步伐。让我们在中共岳阳市委的领导下，抢抓机遇、团结奋进、务实创新，为开创民本岳阳和谐崛起新局面、夺取全面建设小康社会新胜利而努力奋斗！ 小群,2008-01-09 09:06:33

各位代表！历史是人民创造的，任何困难都不能改变岳阳人民创造美好幸福生活的坚定信念，任何挑战都无法阻挡我们实现富民强市目标的前进步伐。让我们在中共岳阳市委的领导下，抢抓机遇、团结奋进、务实创新，为开创民本岳阳和谐崛起新局面、夺取全面建设小康社会新胜利而努力奋斗！
